# A giant intradiploic angiolipoma of the skull: controversies in diagnosis of skull bone lesions.Report of the second case reported in the literature and review of the literature

**Published:** 2012-11

**Authors:** Babak Ghassemi, Abbas Amirjamshidi

**Affiliations:** ^a^Arad General Hospital, Tehran University of Medical Sciences, Tehran, Iran.

## Abstract

The present study reports a very rare case of giant intradiploic angiolipoma. To our knowledge this is the second case reported of an intradiploic angiolipoma that occurred in the calvarium. We present a short controversial discussion regarding the new aspects in differential diagnosis of skull mass lesions according to the findings of this very rare case.

**Keywords:**

Skull, Bone, Lesions


					Volume 4, Suppl. 1 Nov 2012
Publisher: Kermanshah University of Medical Sciences
URL: http://www.jivresearch.org
Copyright:
Congress of Iranian Neurosurgeons, 14-16 November 2012, Kermanshah, Iran
Paper No. 40
A giant intradiploic angiolipoma of the skull: controversies in
diagnosis of skull bone lesions.
Report of the second case reported in the literature and review of the literature
Babak Ghassemi a
, Abbas Amirjamshidi a,*
a
Arad General Hospital, Tehran University of Medical Sciences, Tehran, Iran.
Abstract:
The present study reports a very rare case of giant intradiploic angiolipoma. To our knowledge this is the second
case reported of an intradiploic angiolipoma that occurred in the calvarium. We present a short controversial
discussion regarding the new aspects in differential diagnosis of skull mass lesions according to the findings of this
very rare case.
Keywords:
Skull, Bone, Lesions
* Corresponding Author at:
Abbas Amirjamshidi: Arad General Hospital, Tehran University of Medical Sciences, Tehran, Iran. Tel: 09121329864,
Email: abamirjamshidi@yahoo.com, (Amirjamshidi A.).

				

